# Stability of Antimicrobials in Elastomeric Pumps: A Systematic Review

**DOI:** 10.3390/antibiotics11010045

**Published:** 2021-12-30

**Authors:** Beatriz Fernández-Rubio, Paula del Valle-Moreno, Laura Herrera-Hidalgo, Alicia Gutiérrez-Valencia, Rafael Luque-Márquez, Luis E. López-Cortés, José María Gutiérrez-Urbón, Sonia Luque-Pardos, Aurora Fernández-Polo, María V. Gil-Navarro

**Affiliations:** 1 Unidad de Gestión Clínica de Farmacia, Hospital Universitario Virgen del Rocío, 41013 Seville, Spain; beatrizfernandezrub@gmail.com (B.F.-R.); pauladelvallem.0876@gmail.com (P.d.V.-M.); 2 Unidad de Gestión Clínica de Farmacia, Instituto de Biomedicina de Sevilla (IBiS), Hospital Universitario Virgen del Rocío, 41013 Seville, Spain; mariav.gil.sspa@juntadeandalucia.es; 3 Infección por el VIH y Farmacocinética de Antivirals, Instituto de Biomedicina de Sevilla (IBiS), 41013 Seville, Spain; alicia.gutierrez.valencia@gmail.com; 4 Unidad de Gestión Clinica de Enfermedades Infecciosas, Microbiología y Medicina Preventiva, Instituto de Biomedicina de Sevilla (IBiS), Hospital Universitario Virgen del Rocío, 41013 Seville, Spain; rafaeluquemarquez@gmail.com; 5 Unidad de Gestión Clínica de Enfermedades Infecciosas, Microbiología y Medicina Preventiva, Instituto de Biomedicina de Sevilla (IBiS), Hospital Universitario Virgen Macarena/CSIC/, 41013 Seville, Spain; luiselopezcortes@gmail.com; 6 Unidad de Gestión Clínica de Farmacia, Complexo Hospitalario Universitario de A Coruña, 15006 A Coruna, Spain; Jose.Gutierrez.Urbon@sergas.es; 7 Unidad de Gestión Clínica de Farmacia, Hospital del Mar, 08003 Barcelona, Spain; sluque@psmar.cat; 8 Servicio de Farmacia, Proa-NEN, Hospital Infantil, Vall d’Hebron Barcelona Hospital Campus, 08035 Barcelona, Spain; aufernan@vhebron.net

**Keywords:** antimicrobials, elastomer, OPAT, systematic review

## Abstract

Outpatient parenteral antimicrobial therapy (OPAThttp) programs have become an important healthcare tool around the world. Portable elastomeric infusion pumps are functional devices for ambulatory delivery of antimicrobial drugs, and their stability is an essential point to guarantee an appropriate infusion administration. We conducted a systematic review to provide a synthesis and a critical evaluation of the current evidence regarding antimicrobial stability in elastomeric pumps. Data sources were PubMed, EMBASE, and Web of Sciences. The review protocol was registered on the Center for Open Science, and it was carried out following the PRISMA guidelines. Studies were eligible if the aim was the evaluation of the physicochemical stability of an antimicrobial agent stored in an elastomeric device. Of the 613 papers identified, 33 met the inclusion criteria. The most studied group of antimicrobials was penicillins, followed by cephalosporins and carbapenems. In general, the stability results of the antimicrobials that have been studied in more than one article agree with each other, with the exception of ampicillin, flucloxacillin, and ceftazidime. The antibiotics that displayed a longer stability were glycopeptides and clindamycin. Regarding the stability of antifungals and antivirals, only caspofungin, voriconazole, and ganciclovir have been investigated. The information provided in this article should be considered in patient treatments within the OPAT setting. Further stability studies are needed to confirm the appropriate use of the antimicrobials included in this program to ensure optimal patient outcomes.

## 1. Introduction

Over the last several decades, outpatient parenteral antimicrobial therapy (OPAT) programs have been implemented as a useful healthcare tool worldwide. These programs enable clinically stable patients to receive optimal antimicrobial treatment after hospital discharge [1]. There are two modalities of OPAT programs, one of which involves the administration of the antimicrobial in the patient’s home and the other at an infectious disease outpatient center [2]. OPAT programs provide several advantages, such as the improvement of the quality of life of patients, allowing them to continue with their daily routine at home despite receiving intravenous antibiotics. Additionally, they reduce the potential risk of acquiring nosocomial infections or hospital-acquired delirium in elderly patients, and they decrease the mean number of hospitalization days, which is beneficial for the healthcare system [3]. Nevertheless, current OPAT models have some limitations, including the potential administration-related toxicity and the requirement of a healthcare professional in order to set up the pumps [4], especially if the administration is carried out at the outpatient facility.

Portable elastomeric infusion pumps are functional devices for the delivery of intravenous drugs, focused on making the administration easier [5]. They are lightweight devices with a transparent plastic container, inside which there is an elastomeric reservoir, commonly made of latex, polyisoprene, or silicone, which contains the medication. Its operation is based on the elastomeric property of the balloon to release the drug solution at a constant flow along an infusion line [6]. These pumps have undergone significant development, with a rising frequency of use due to the advantages that they offer [7]. The main qualities include portability, non-electronic handling, and ease of management by the patient or a relative, providing more autonomy to the patient. Furthermore, elastomeric pumps allow a safe, continuous drug administration at a constant flow rate and in absolute silence, and they have a better cost effectiveness profile than electronic models [8]. However, the use of elastomeric pumps involves some limitations, including a lower delivery rate accuracy than electronic pumps, the necessity to choose a specific speed through the whole administration, and the lack of an alarm if a failure occurs during the drug delivery. In addition, environmental factors such as external temperature need to be taken into account, as this could affect the stability of antibiotics [9,10].

In this context, antimicrobial stability is an essential point to guarantee an optimal health outcome [11]. Conditions affecting stability are varied, including the concentration of the drug, diluents and additives used; the temperature and duration of the storage; and the composition of the elastomeric device used. Both chemical stability (percentage of the drug that remains after the storage) and physical stability (changes in color or clearness, pH, and particle formation) should be studied. Therefore, stability data are essential requirements for safety management [12,13]. The aim of this systematic review is to provide a synthesis and a critical evaluation of the current evidence regarding antimicrobial stability in elastomeric pumps.

## 2. Results

**Figure 1:**
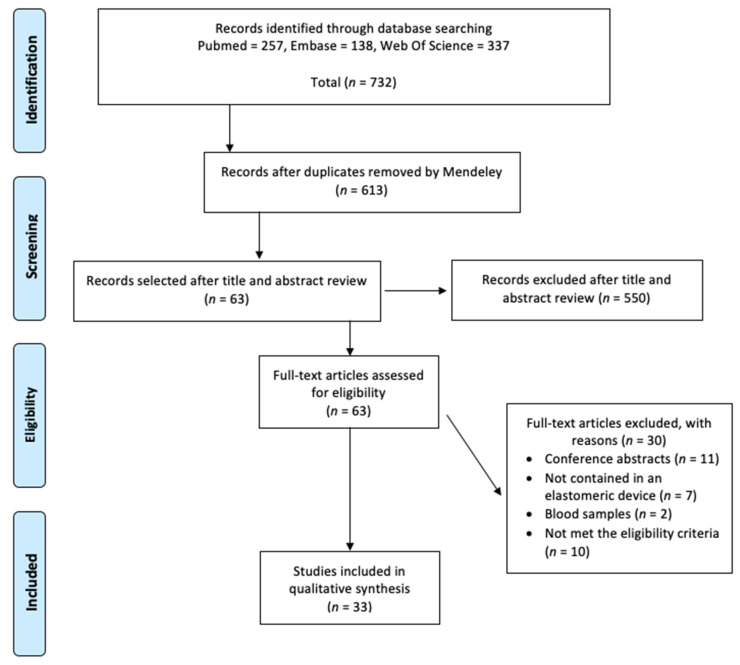
Study selection flowchart.

The initial search found 613 papers after duplicates were removed. Following preliminary title and abstract review, 63 records were selected for full-text review. Thirty papers were excluded because eleven papers were published in a conference abstract format, seven were not contained in an elastomeric device, two measured the stability within blood samples, and ten did not evaluate the physicochemical stability of the antimicrobial. Finally, 33 articles were included in the systematic review (Figure 1).

The principal characteristics of each antimicrobial group, such as drug, reference [14,15,16,17,18,19,20,21,22,23,24,25,26,27,28,29,30,31,32,33,34,35,36,37,38,39,40,41,42,43,44,45,46], main conditions (composition of the elastomeric device chosen, concentration, diluent, temperature, and duration of storage), chemical and physical stability obtained, and the most relevant comments for the systematic review, are summarized in Table 1, Table 2, Table 3, Table 4, Table 5, Table 6, Table 7 and Table 8.

The most studied group of antimicrobials has been the penicillins. Within the group, amoxicillin, flucloxacillin, and piperacillin/tazobactam have had the most elastomer stability studies performed (Table 1). Their maximum stability is just 24 h, though this depends greatly on the temperature, and some exceptions can be found in the case of amoxicillin. Regarding the stability of cephalosporins, cefazolin and ceftazidime are the most commonly used in elastomeric devices, since they are stable in both normal saline and 5% dextrose in a wide range of concentrations under different storage conditions (Table 2). Additionally, the composition of the elastomeric device can be latex, silicone, or polyisoprene. Meropenem is the most studied carbapenem, including the combination with vaborbactam, a beta-lactamase inhibitor. In the studies performed with meropenem alone, both experiments proved that the stability is highly dependent on the concentration, since it decreases if the concentration is raised under all storage conditions (Table 3). In relation to aminoglycosides, glycopeptides, and other antibiotics (clindamycin and colistin), their physicochemical stability is variable (Table 4, Table 5 and Table 6, respectively). With the exception of aminoglycosides, the rest of the antibiotics have shown a long stability contained in elastomeric devices. Regarding the stability of antifungals, just voriconazole and caspofungin have been investigated (Table 7). The stability of caspofungin is longer than that of voriconazole. However, this depends on the composition of the elastomeric device. In relation to antivirals, only ganciclovir was studied in a single paper (Table 8).

## 3. Discussion

Considering that elastomeric devices have shown their great utility in OPAT [47], this systematic review provides a comprehensive overview of antimicrobial stability, both physical and chemical, in elastomeric pumps.

Stability in elastomers has been mainly studied in antibiotics, compared to the experiments published of antivirals and antifungals. Penicillins (in particular amoxicillin, flucloxacillin, and piperacillin/tazobactam) and cephalosporins (especially ceftazidime) are the groups of antibiotics that stand out. In general, the stability results of the antimicrobials that have been studied in more than one article agree with each other. However, some exceptions can be found in the case of ampicillin [14,15], flucoxacillin [20,21,22,23], and ceftazidime [14,30,33,34]. Regarding ampicillin, the conditions for each of the experiments were different in terms of their elastomer composition, concentration, and diluent. Therefore, it is difficult to draw firm conclusions. In the case of ceftazidime, results also differ between the studies, but chemical instability of this antibiotic seems to occur when the concentration is high (120 mg/mL). On the contrary, a lack of stability of flucloxacillin in elastomers is related to high temperatures. Additionally, it should be remarked that, while chemical stability is reflected in the results of all the studies, physical stability is only included in a few of them, with the changes in pH and color being the most studied.

The composition of the elastomeric reservoir varied between the studies, although the most prevalent has been polyisoprene, followed by latex and silicone. Polyisoprene is a synthetic polymer that provides many of the same properties as natural rubber latex without the latex allergen concerns. Both polymers offer good resistance to most alcohols, acids, bases, and polar solvents and are well-suited for low-temperature environments. In relation to silicone, it is a material that is non-reactive, stable, and resistant to extreme environments and temperatures while still maintaining its useful properties [48,49]. There are no published studies that establish a relationship between the material of the elastomeric pump and drug stability. However, one of the studies included in the review has found that, depending on the polymer used, the stability varies [45]. Therefore, it is a factor to take into account in future stability studies. Additionally, it should be noted that, in the case of widely used antibiotics such as vancomycin [14,30] or ceftriaxone [14,30], no published study has analyzed their stability in polyisoprene elastomers. Furthermore, some antimicrobials useful in the OPAT setting have not been studied contained in elastomeric devices. Among these antimicrobials, teicoplanin is particularly relevant, since glycopeptides, along with beta lactams, are the most frequently used antibiotics in OPAT programs [50]. Furthermore, there is a lack of stability studies in elastomers of antimicrobials such as daptomycin [51,52] or amphotericin b [53,54], although this antifungal, along with fluconazole, anidulafungin and micafungin, is used in OPAT programs.

The main strength of this analysis is that it is the first systematic dealing with the physical and chemical stability of antimicrobials in elastomeric devices. Recently, two reviews related to the stability of antimicrobials in elastomers have been published. The first one [55] updates the data from a previous study [56] that was carried out to find out if the stability of antimicrobials in elastomeric pumps meets the quality standards of the Yellow Covered Document (YCD) from the UK National Health System. The second one [57] focuses on evaluating the different means of using elastomeric infusion pumps in out-of-hospital administration of intravenous antibiotics. Therefore, there is no systematic review that provides information on the physicochemical stability of antimicrobials in elastomers together.

This systematic review has some limitations: the analytical techniques used in the different studies have not been described, so there could be certain differences in the methodology of the experiments that have not been analyzed. In addition, this study was limited to including only peer-reviewed articles, so there may be further gray literature supporting the stability of some of the drugs mentioned.

## 4. Materials and Methods

The review protocol was registered on the Center for Open Science (DOI number: 10.17605/OSF.IO/Y2KJV), and it was carried out following the main criteria of the Preferred Reporting Items for Systematic Reviews and Meta-Analyses (PRISMA) Equity 2012 Extension declaration [58].

### 4.1. Eligibility Criteria

We selected the studies that met the following inclusion criteria:

1. The study drug was an antimicrobial agent, including antibiotics, antivirals, and antifungals.
2. The aim of the study was the evaluation of the physicochemical stability of the antimicrobial agent stored in an elastomeric device.

We dismissed the studies that met the following exclusion criteria:

3. Studies that evaluated admixtures of multiple drugs in the same solution.
4. Studies that measured the serum disposition of the antimicrobial agent in patients.
5. Conference abstracts.

### 4.2. Information Sources

An electronic literature search was performed using MEDLINE (through PubMed interface), EMBASE, and Web of Science Core Collection databases on 1 July 2020, with no publication date restrictions. The search strategy was composed of MeSH terms and free text (keywords and synonyms) combined with Boolean operators. The search strategy was arranged according to each database. Additionally, the reference lists of selected studies were hand-searched to identify any other relevant studies. The search strategy is detailed in Table 9.

### 4.3. Study Selection

Two independent reviewers (B.F.-R. and P.d.V.-M.) screened the titles and abstracts of all eligible publications for possible inclusion after duplicate removal. To ensure inter-rater reliability, 100% of the articles were assessed independently by both authors. The articles included were read at their full length before a final decision on inclusion. Any disagreement was settled by consensus with a third reviewer (L.H.-H.).

### 4.4. Data Collection and Analysis

Two reviewers (B.F.-R. and P.d.V.-M.) independently extracted data, and L.H.-H. examined all extraction sheets to ensure their accuracy. We explicitly stated whether there were any data missing from the studies. For each publication, the following variables were registered:

1. Antimicrobial drug studied.
2. Author details and year of publication.
3. Conditions:
  ○ Composition of the elastomeric device used.
  ○ Concentration of the drug studied.
  ○ Diluent used.
  ○ Temperature and duration of storage.
4. Chemical stability demonstrated under each condition: concentration of all samples remained higher than 90% of the original concentration.
5. Physical stability demonstrated under each condition: particle formation, changes in color or clearness, and pH analysis.
6. Comments, included buffers of other additives used.

## 5. Conclusions

The information provided in this systematic review should be considered in the treatment of patient within the OPAT setting. Nevertheless, further stability studies are needed to confirm the appropriate use of the antimicrobials included in this program to ensure optimal patient outcomes.

## Figures and Tables

**Table 1:** Stability of penicillins in elastomeric devices.

Drug	Reference	Composition of the Elastomeric Device	Concentration (mg/mL)	Diluent	Temperature and Duration of Storage	Chemical Stability	Physical Stability	Comments
Ampicillin	[14]	Latex	20	NS	5 °C: 3 d 25 °C: 8 h	Yes	Not studied	
	[15]	Polyisoprene	50	Acetate ringer solution	4 °C: 10 d 25 °C: 24 h 31.1 °C: 24 h	No	Not studied	
Amoxicillin	[16]	Polyisoprene	25	NS	5 °C: 48 h	Yes	Not studied	Also measured effectiveness of plasma amoxicillin concentrations
	[17]	Polyisoprene	20, 40, and 60	NS	20 °C: 48 h 25 °C: 48 h	Yes (only at 20 and 40 mg/mL)	Yes (not precipitate or color changes)	No chemical stability at 60 mg/mL
	[18]	Polyisoprene or silicone	25, 50, 125, and 250	Sterile water	5 °C: 24 h 25 °C: 12 h	Yes (just the lowest concentration, 25 mg/mL)	Not studied	No chemical stability at high concentrations and at high temperatures (more than 30 °C)
	[19]	Polyisoprene	25, 50, and 83.3	NS	4 °C: 48 h 25 °C: 48 h	Yes	Not studied	
Benzylpenicillin	[15]	Polyisoprene	100,000 units/mL	Acetate ringer solution	4 °C: 10 d 25 °C: 24 h 31.1 °C: 24 h	Yes	Not studied	
Flucloxacillin	[20]	Silicone and polyisoprene	10 and 50	0.3% *w*/*v*	5 °C: 14 d, then 24 h at 32 °C	Yes	Not studied	Citrate-buffered saline pH 7
	[21]	Polyisoprene	50	NS	5 °C: 6 d 5 °C: 6 d, then 24 h at 31 °C	Yes	Not studied	It was not chemically stable when the temperature was raised to 37 °C for 7 h after 6 d at 5 °C and 24 h at 31 °C
	[22]	Polyisoprene	50	NS or water for injection with or without phosphate buffer (0.384 M; pH 7)	4 °C: 6 d 4 °C: 6 d, then 24 h at 37 °C.	Yes	Not studied	At 37 °C, the unbuffered solution was not chemically stable
	[23]	Polyisoprene	33	NS	26.2 °C: 24 h 30.9 °C: 24 h	No	Not studied	Study under real-life situations
Mezlocillin	[14]	Latex	20	5D	5 °C: 7 d 20 °C: 4 w 25 °C: 48 h	Yes	Not studied	
Nafcillin	[14]	Latex	20	NS or 5D	−20 °C: 12 w 5 °C: 4 d 25 °C: 24 h	Yes	Not studied	
Penicillin G sodium	[24]	Polyisoprene	2500 and 50,000 units/mL	NS or 5D	5 °C: 21 d	Yes	No (pH consistently decreased, from 6.4 to 5.5; no change in appearance)	After 28 d, 2500 units/mL with NS was not chemically stable
Piperacillin	[14]	Latex	30	NS or 5D	−20 °C: 4 w 5 °C: 7 d 25 °C: 24 h	Yes	Not studied	
Piperacillin/ tazobactam	[25]	Polyisoprene	67/8	NS or 5D	31.1 °C: 24 h	Yes	Yes (pH)	
	[26]	Polyisoprene	9/1.15 50/6.2 90/11.25	NS	35 °C: 72 h	No	No (pH changed although not precipitate or color changes)	
	[27]	Polyisoprene	22/3 80/10	NS	5 °C: 13 d, then 24 h at 32 °C	Yes	Not studied	Use of a citrate-buffered saline diluent pH 7
	[23]	Polyisoprene	50/6.25	NS	26.2 °C: 24 h 30.9 °C: 24 h	Yes	Not studied	Study under real-life situations
Temocillin	[28]	Polyisoprene	10 and 20	Water for injection	4 °C: 4 w 4 °C: 4 w, then 24 h at 25 °C	Yes	Not studied	

NS: normal saline, 5D: 5% dextrose, h: hours, d: days, w: weeks.

**Table 2:** Stability of cephalosporins in elastomeric devices.

Drug	Reference	Composition of the Elastomeric Device	Concentration (mg/mL)	Diluent	Temperature and Duration of Storage	Chemical Stability	Physical Stability	Comments
Cefazoline	[14]	Latex	20	NS or 5D	−20 °C: 12 w 5 °C: 7 d 25 °C: 24 h	Yes	Not studied	
	[25]	Polyisoprene	25	NS or 5D	31.1 °C: 24 h	Yes	Yes (pH)	
	[29]	Polyisoprene	12.5 and 25	NS or 5D	4 °C: 72 h, then stored at 35 °C for 12 h, followed by 25 °C for 12 h.	Yes	Yes (pH unchanged, clear/no haziness, no particles).	
	[23]	Polyisoprene	25	NS	26.2 °C: 24 h 30.9 °C: 24 h	Yes	Not studied	Study under real-life situations
	[30]	Silicone	5 and 40	NS or 5D	4 °C: 26 d 23 °C: 3 d	Yes	Not studied	
Cefepime	[23]	Polyisoprene	12.5	NS	26.2 °C: 24 h 30.9 °C: 24 h	Yes	Not studied	Study under real-life situations
Cefmetazole	[25]	Polyisoprene	33	NS or 5D	31.1 °C: 24 h	Yes	Yes (pH)	
Ceftaroline	[31]	Polyisoprene	6	NS or 5D	4 °C: 44 h 25 °C: 24 h 30 °C: 12 h 35 °C: 12 h with NS and 6 h with 5D	Yes	Yes (no particle formation, color change, or pH change)	
	[32]	Polyisoprene or silicone	12	NS or 5D	2 °C–8 °C: 24 h, then 6 h at 25 °C.	Yes	Yes (clear, colorless, and free of visible particulates; no major change in pH)	
Ceftazidime	[14]	Latex	20	NS or 5D	−20 °C: 12 w 5 °C: 7 d 25 °C: 18 h	Yes	Not studied	
	[33]	Polyisoprene	60 and 120	NS	4 °C: 48 h, then 27 °C for 24 h. 4 °C: 144 h, then 27 °C for 24 h. 27 °C: 24 h.	No	Yes (clear, colorless, and free of visible particulates)	
	[34]	Polyisoprene	60	NS	–20 °C: 14 d 4 °C: 14 d	Yes	Not studied	The degradation product pyridine was detected at all storage times
	[30]	Silicone	NS: 5 and 60 5D: 5 and 40	NS or 5D	23 °C: 1 d 4 °C: 4 d	Yes	Not studied	
Ceftolozane-tazobactam	[35]	Polyisoprene	1.25/0.63 12.5/6.25 25/12.5	NS	4 °C: 7 d 25 °C: 24 h 37 °C: 24 h	Yes	Not studied	Tazobactam was more stable than ceftolozane
	[36]	Polyisoprene	1 g/0.5 g 100 mg/50 mg	NS or 5D	5 °C: 10 d 25 °C: 24 h	Yes	Yes (clear and free of visible particulates; no changes in pH)	
Ceftriaxone	[14]	Latex	20	NS or 5D	−20 °C: 26 w 5 °C: 10 d 25 °C: 3 d	Yes	Not studied	
	[30]	Silicone	5 and 40	NS or 5D	4 °C: 14 d 23 °C: 2 d	Yes	Not studied	

NS: normal saline, 5D: 5% dextrose, h: hours, d: days, w: weeks.

**Table 3:** Stability of carbapenems in elastomeric devices.

Drug	Reference	Composition of the Elastomeric Device	Concentration (mg/mL)	Diluent	Temperature and Duration of Storage	Chemical Stability	Physical Stability	Comments
Doripenem	[25]	Polyisoprene	12.5	NS or 5D	31.1 °C: 24 h	No	Yes (pH)	
	[37]	Polyisoprene	5 and 10	NS or 5D	−20 °C: 28 d 4 °C: 10 d in NS and 7 d in 5 d 25 °C: 24 h in NS and 16 h in 5D	Yes	No	A white precipitate, which returned to solution by shaking, was noted after thawing the frozen containers
Ertapenem	[38]	Polyisoprene	10	NS	5 °C: 72 h	Yes	Not studied	
Imipenem- cilastatin	[14]	Latex	5	NS or 5D	5 °C: 1 d 25 °C: 4 h	Yes	Not studied	
Meropenem	[25]	Polyisoprene	12.5	NS or 5D	31.1 °C: 24 h	No	Yes (pH)	
	[39]	Polyisoprene	6, 12, 20, and 25	NS	5 °C: 6 d 5 °C: 48 h, then 4 d at 25 °C	Yes (just the lowest concentration, 6 mg/mL)	Yes (pH)	At higher concentrations (25 mg/mL), no chemical stability
	[40]	Polyisoprene	4, 10, and 20	NS	5 °C: 5 d	Yes	Not studied	The lowest concentration (4 mg/mL) showed chemical stability for 7 d
Meropenem/ vaborbactam	[41]	Polyisoprene	5.7/5.7	NS	4 °C: 120 h 24 °C: 12 h	Yes	Not studied	

NS: normal saline, 5D: 5% dextrose, h: hours, d: days.

**Table 4:** Stability of aminoglycosides in elastomeric devices.

Drug	Reference	Composition of the Elastomeric Device	Concentration (mg/mL)	Diluent	Temperature and Duration of Storage	Chemical Stability	Physical Stability	Comments
Gentamicin	[14]	Latex	0.8	NS	25 °C: 24 h	Yes	Not studied	
Tobramycin	[14]	Latex	0.8	NS	25 °C: 24 h	Yes	Not studied	

NS: normal saline, h: hours.

**Table 5:** Stability of glycopeptides in elastomeric devices.

Drug	Reference	Composition of the Elastomeric Device	Concentration (mg/mL)	Diluent	Temperature and Duration of Storage	Chemical Stability	Physical Stability	Comments
Telavancin	[42]	Polyisoprene	0.6 and 8.0	NS, 5D, orsterilized water	5 °C: 8 d	Yes	Yes (pH)	Sterilized water (0.6 mg/mL) and NS (8.0 mg/mL) were followed by Ringer’s lactate solution
Vancomycin	[14]	Latex	5	NS or 5D	−20 °C: 9 w 5 °C: 14 d 25 °C: 24 h	Yes	Not studied	
	[30]	Silicone	1 and 5	NS or 5D	4 °C: 27.8 d 23 °C: 7.5 d	Yes	Not studied	

NS: normal saline, 5D: 5% dextrose, h: hours, d: days, w: weeks.

**Table 6:** Stability of other antibiotics in elastomeric devices.

Drug	Reference	Composition of the Elastomeric Device	Concentration (mg/mL)	Diluent	Temperature and Duration of Storage	Chemical Stability	Physical Stability	Comments
Clindamycin	[30]	Silicone	1 and 12	NS or 5D	4 °C: 27.8 d 23 °C: 7.5 d	Yes	Not studied	
Colistin methanesulfonate	[43]	Polyisoprene	2 MU	NS	5 °C: 8 d 22 °C: 8 d	Yes	Yes (pH)	
Colistimethate sodium	[44]	Polyisoprene	0.8	NS	4 °C: 7 d	Yes	Not studied	

NS: normal saline, 5D: 5% dextrose, d: days.

**Table 7:** Stability of antifungals in elastomeric devices.

Drug	Reference	Composition of the Elastomeric Device	Concentration (mg/mL)	Diluent	Temperature and Duration of Storage	Chemical Stability	Physical Stability	Comments
Caspofungin	[45]	Polyisoprene or silicone	0.2, 0.28, and 0.5	NS	5 °C: 14 d 25 °C: 60 h	Yes, in the polyisoprene infuser	Not studied	Not chemically stable in the silicone infuser
Voriconazol	[46]	Polyisoprene	2	NS or 5D	4 °C: 96 h 25 °C: 4 h 35 °C: 4 h	Yes	Not studied	

NS: normal saline, 5D: 5% dextrose, h: hours, d: days.

**Table 8:** Stability of antivirals in elastomeric devices.

Drug	Reference	Composition of the Elastomeric Device	Concentration (mg/mL)	Diluent	Temperature and Duration of Storage	Chemical Stability	Physical Stability	Comments
Ganciclovir	[14]	Latex	5	NS	5 °C: 5 d 25 °C: 24 h	Yes	Not studied	

NS: normal saline, h: hours, d: days.

**Table 9:** Complete search strategy for different databases.

Healthcare Database	Search Strategy
PubMed	(stability) AND (elastomer OR elastomeric) AND (anti-infective agent OR antibiotic OR antimicrobial)
EMBASE	(‘stability’/exp) AND (‘elastomer’/exp OR ‘elastomeric’/exp) AND (‘anti-infective agent’/exp OR ‘antibiotic’/exp OR ‘antimicrobial’/exp)
Web of Science	TS = (stability AND (elastomer OR elastomeric) AND (anti-infective agent OR antibiotic OR antimicrobial))
